# Impact of Selenium Addition to Animal Feeds on Human Selenium Status in Serbia

**DOI:** 10.3390/nu10020225

**Published:** 2018-02-16

**Authors:** Zoran Pavlovic, Ivanka Miletic, Milica Zekovic, Marina Nikolic, Maria Glibetic

**Affiliations:** 1 Institute for Public Health Pozarevac, Jovana Serbanovica 14, 12000 Pozarevac, Serbia; 2 Institute of Bromatology, Faculty of Pharmacy, Vojvode Stepe 450, 11221 Belgrade, Serbia; miletic_i@ikomline.net; 3 Institute for Medical Research, Centre of Research Excellence in Nutrition and Metabolism, University of Belgrade, Tadeusa Koscuska 1, 11000 Belgrade, Serbia; zekovicmilica@gmail.com (M.Z.); marina.nikolic12@yahoo.com (M.N.); mglibetic@gmail.com (M.G.)

**Keywords:** plasma selenium, supplementation of feed, food of animal origin, food frequency questionnaires

## Abstract

Research conducted during the 1980s demonstrated Se deficiency in humans. Increased inclusion of selenium in animal feeds started from the year 2000 onwards. The aim of this study was to estimate the effects of selenium inclusion in animal feeds on human selenium status and dietary habits of the Serbian population related to food of animal origin. Plasma selenium concentration in healthy adult volunteers, including residents of one of the regions with the lowest (Eastern Serbia, *n* = 60) and of one of the regions with the highest Se serum levels reported in the past (Belgrade, *n* = 82), was determined by hydride generation atomic absorption spectrometry. Multivariate analysis was employed to determine the correlation between Se plasma levels and dietary intake data derived from food frequency questionnaires and laboratory tests. The mean plasma Se level of the participants was 84.3 ± 15.9 μg/L (range: 47.3–132.1 μg/L), while 46% of participants had plasma Se levels lower than 80 μg/L. Frequency of meat, egg, and fish consumption was significantly correlated with plasma selenium level (r = 0.437, *p* = 0.000). Selenium addition to animal feed in the quantity of 0.14 mg/kg contributed to the improvement of human plasma selenium levels by approximately 30 μg/L.

## 1. Introduction

Selenium is a trace element essential for humans and animals. Since selenium enters the food chain through incorporation into edible plants, the amount of Se in food and Se status in humans and animals reflects the Se content in the tissue of plants cultivated in the corresponding region.

The geographical Se distribution in soil was found to be inadequate in many regions in the world, including Serbia [1,2]. The assumption is that 0.5–1 billion people worldwide are Se-deficient. Despite the prevalence of Se deficiency, there are limited options available to provide adequate dietary Se intake for the general population, such as the addition of selenium to fertilizers in Finland [3], or the fortification of table salt with Se [4].

Selenium supplementation in animal feeds has been used in livestock production in the last 40 years with the aim of improving the health and production performance of animals. Furthermore, the inclusion of Se in animal feed resulted in increased Se content in meat, eggs, and milk [5,6,7]. Although Se supplementation in animal husbandry is undoubtedly the most important activity for the general improvement of Se status in humans, at the moment, there are no data about the magnitude of that influence. Research conducted in the 1990s showed that the low content of Se in Serbian soil was associated with deficient Se contents in feed and food, as well as with Se deficiency in humans [8,9]. Selenium feed supplementation in Serbia started from the year 2000 onwards, and, as a consequence, selenium levels in food originating from farmed animals have been raised [7]. Given that food of animal origin holds an important place in Serbian nutrition, we hypothesize that the improvement of selenium status in a healthy Serbian population, reported from the year 2000 onwards [10], could be the result of the increased inclusion of selenium in animal feed. Furthermore, apart from the mandatory selenium supplementation of feed, during the last 25 years in Serbia there have not been other activities that could influence the dietary selenium intake of the general population, providing a unique opportunity for the estimation of the impact of selenium addition to animal feeds on public health.

Our study had two main objectives: first, to estimate the effects of selenium inclusion in animal feed on human selenium status, and second, to determine the correlations between plasma selenium level and dietary habits regarding food of animal origin.

## 2. Materials and Methods

### 2.1. Participants and Data Collection

According to the data of Maksimovic and coworkers [8], one of the regions with the lowest human Se serum concentrations (East Serbia) and one with the highest (Belgrade) were selected. Blood samples were obtained from healthy adult volunteers of both genders, including 82 Belgrade residents and 60 East Serbia residents of Pozarevac City and its surroundings. Ethical approval for the study was obtained from the Clinical Hospital Center Zemun (Serbia) Ethics Committee, reference number No 2125. All participants gave written informed consent for inclusion before they participated in the study. Data related to age, weight, height, and individual lifestyle (alcohol consumption, smoking habits, physical exercise, and supplement intake) were collected using standardized questionnaires. A comprehensive food frequency questionnaire (FFQ), validated for the assessment of vitamin D intake of people in Serbia, was adapted and used to assess dietary practice and level of animal origin food consumption [11]. Nutrient intake resulting from the FFQ was estimated using the Serbian Food Composition Database [12] and DIETS ASSESS & PLAN software tool, which was used and validated in numerous national and regional food consumption surveys and evaluated by the European Food Safety Authority’s project [13]. Body Mass Index (BMI) was calculated as weight in kilograms divided by the square of height in meters (kg/m^2^).

Fasting blood samples were drawn in the morning by venipuncture in Vacutainer tubes containing sodium heparin as an anticoagulant. Heparinized blood was centrifuged (3000 rpm, 15 min) for the preparation of plasma, and aliquots of plasma were stored at −20 °C until analysis.

### 2.2. Laboratory Assays

Plasma selenium concentrations were measured by hydride generation atomic absorption spectrometry (HG-AAS) on a Varian SpectrAA-10 instrument with a VGA-76 hydride unit, using the following instrument parameters: wavelength 196.0 nm, slit width 1.0 nm, reductant 0.6% NaBH_4_ in 0.5% NaOH, and acid 10 M HCl. Samples were mineralized according to the method reported by Yu et al. [14]. To verify the accuracy of the method, control serum ClinChek-Control (Recipe, Chemical + Instruments Gmbh, Germany) with selenium contents of 62.3 ± 12.7 µg/L (Level I) and 103 ± 20.8 µg/L (Level II) was analyzed. Method performances were monitored by analysis of the same control serum in each series. The obtained results for Se content in the control serums were 64.5 ± 6.9 µg/L (Level I) and 101.5 ± 9.3 µg/L (Level II), which agree with the certified value. 

### 2.3. Statistical Analyses

General characteristics of the study population and selenium status are presented as means for continuous variables and percentages for categorical variables. The Kruskal-Wallis-Wilcoxon test was used to test the equality of the mean values of food intake frequency for the population grouped by quartiles of plasma selenium concentrations. Spearman’s rank correlation coefficient was used to evaluate the relationship between the frequency of animal origin food intakes and plasma selenium level. The significance level for all analyses was set at *p* < 0.05. Statistical analyses of results were made with the use of SPSS version 11.5.

## 3. Results

Characteristics of the study population are shown in Table 1. A total of 142 subjects were recruited (81 women and 61 men) with a mean age of 41.2 ± 8.4 years, ranging between 22 and 63 years. The mean plasma Se level of the participants was 84.3 ± 15.9 μg/L, ranging between 47.3 and 132.1 μg/L. Based on chemical analyses, 46% of the participants had a plasma Se level lower than 80 μg/L. There were no significant differences in plasma Se level between residents of Belgrade (84.1 ± 17.9 μg/L) and East Serbia regions (84.8 ± 11.9 μg/L). Furthermore, no significant difference in plasma Se levels existed between sexes, nor between people grouped by age, BMI, smoking habits, education, or use of vitamin and mineral supplements.

Data obtained from the food frequency questionnaire are summarized in Table 2. All subjects consumed food of animal origin at least once a day and one quarter of the study population consumed animal products at least twice a day. The most common animal-based foods in Serbian diet are milk and dairy products, followed by meat. The frequency of meat consumption had a significant, moderate strong, and positive correlation with plasma Se (Spearman’s rank correlation coefficient r = 0.369, *p* = 0.000). On the other side, fish is rarely present in the diet. More than one third of subjects consumed fish less than once per week. Similarly, one third of the population consumed eggs less than once per week, and one half consumed eggs 1 to 2 times per week.

## 4. Discussion

The mean plasma Se level observed in this study was in accordance with recent results from Serbia [10], as well as with results reported for healthy individuals of similar age in France [15], Italy [16], and Great Britain [17]. Serum selenium concentrations observed in studies conducted in neighboring countries were lower or similar to the Serbian data: Hungary 77.4 ± 14.82 μg/L [18], Croatia 66.8 ± 14.43 μg/L [19], and Bulgaria 84.82 ± 1.18 μg/L [20].

There are no established universal normal ranges of plasma or serum selenium levels, because of the variability in blood selenium levels caused by differences in soil selenium levels, dietary habits, use of selenium supplements in animal feed, and consumption of certain specific imported foods such as wheat. For the assessment of deficiency or adequacy of human selenium status, one of the criteria is based on blood or plasma selenium concentrations, at which maximal activities of selenoproteins-glutathione peroxidase and selenoprotein P are observed. The plasma selenium concentration needed to achieve a full expression of selenoproteins is about 80 to 90 µg/L [21]. The mean plasma selenium of the studied population was near the middle of the range, but it should be noted that 46% of participants had plasma selenium levels below the lowest threshold for the maximal activity of selenoproteins. Therefore, further activities and targeted public health initiatives directed towards the improvement of selenium status among Serbian residents are necessary.

The association between plasma selenium and various factors like gender, age, smoking, and BMI was the subject of extensive research. However, due to the disagreement on reported data, it is difficult to draw conclusions. In this study, no significant differences between males and females in plasma Se concentrations were observed. These findings were in accordance with the majority of data reported in scientific articles describing populations with similar selenium status [7,22,23,24]. Concerning populations with higher selenium levels, other authors have found significantly higher serum Se concentrations in males when compared to females [25,26,27]. Certain studies demonstrated the association between plasma Se levels and age [17,26,28], while our results correspond to the results of other authors [15,16,22,23,25,27], who were not able to find a significant correlation between selenium status and age. There is evidence that selenium status is inversely associated with the prevalence of smoking [16,17,26,27,28]. However, our results, similar to those of Chen and coworkers [22] and Yang and coworkers [23], indicate no correlation between selenium status and smoking habits. Body mass index remains the factor without a clear influence on selenium status. There was no significant association of plasma selenium with BMI observed in this study, which agrees with recently published data [15,16,17,25,28], while other authors reported a negative correlation of selenium status with BMI [23,26,27]. A relatively small sample size is the major limitation of this study, and we speculate that it might be the possible reason for the absence of statistically significant differences. Even though this paper provided valuable data for public health surveillance of selenium adequacy in Serbia, further research with a larger number of participants is needed for additional exploration and better understanding of these relationships.

Food of animal origin is, traditionally, of great importance to the dietary pattern common in Serbia. The average annual consumption of animal origin food is 58 kg meat (as meat and meat products), 160 L milk (as milk and dairy products), and 220 eggs [29]. The dietary practice of the observed population, assessed by a food frequency questionnaire, corresponded to data obtained from the Statistical Office of the Republic of Serbia [29]. The discrepancy observed in egg intake might be due to the fact that participants reported only intake of eggs as is, and that the culinary use of eggs was not counted. In previous research we found that the dietary selenium intake in the Serbian population is insufficient, only 40.9 µg per day, and that food of animal origin presents the greatest contribution to dietary selenium intake total, of 31.7 µg Se/day [7]. Data obtained in this study also corroborate the importance of the consumption of such foodstuffs for human selenium intake in Serbia. The positive correlation of the dietary intake frequency of meat with plasma Se observed in this study agrees with the findings in other research [24,30].

The first large-scale research regarding the Se status of the Serbian population, which started in 1988, showed Se deficiency among the population, with mean Se serum concentrations of 44.2 µg/L, which was among the lowest reported in Europe [8]. Inclusion of selenium in animal feed in Serbia started in 1989, but only for some categories of farm animals [7]. Since 2000, the minimum content of selenium in animal feed prescribed by law is 0.1 mg/kg for pigs and cows and 0.15 mg/kg for poultry [7]. In our previous paper [7], we reported the average selenium supplementation of feed in Serbia to be a quantity of 0.14 mg Se/kg. Furthermore, compared to 1991 [9], increased Se content of meat and eggs was found (Table 3). The implementation of selenium inclusion to feed from 2000 onwards also coincides with data concerning the improvement of human Se status in Serbia obtained by this study. Aside from changes in the feed industry, there were not other activities in Serbia that would influence dietary selenium intake of the general population. Hence, we assume that the supplementation of 0.14 mg Se/kg in animal feed is the predominant reason for the improvement of Se status in the Serbian population up to about 30 μg/L.

Selenium addition to animal feed in Serbia is mostly (92%) in the form of the inorganic Se compound, sodium selenite (SS), followed by an organic form, selenized yeast (SY) [7]. Both inorganic and organic Se forms result in an increased Se content in meat, eggs, and milk but SY is more effective than SS [5,6,31], so the substitution of SS with SY in animal feed could be a simple and fast method to further increase the selenium content in animal foodstuffs, and thus improvement the health of Serbian citizens.

The addition of selenium to fertilizers in agriculture is another way for selenium to enter the food chain. Systematic nationwide supplementation of fertilizers with sodium selenate in Finland started in 1985, resulting in a Se concentration increase in food of both plant and animal origins [3]. Compared to 1985, the present average dietary human intake has doubled and the mean human plasma Se concentration increased from 0.89 μmol/L to a level of 1.40 μmol/L [3]. Finnish and Serbian supplementation examples represent a well-controlled way to increase human Se dietary intake.

## 5. Conclusions

The findings of this study suggest that the implementation of mandatory selenium fortification of fodder has proven its efficacy as a public health strategy directed toward promoting selenium adequacy. Selenium addition to feed in a quantity of 0.14 mg/kg contributed to the improvement of human plasma selenium levels by approximately 30 μg/L for a population with an average annual consumption of 58 kg meat (as meat and products), 160 L milk (as milk and dairy products), and 220 eggs per person. However, continuous monitoring and further research are necessary to draw definitive causal inference. Additional strategies and novel approaches, such as adjustment of the quantity of Se in animal feed or the replacement of SS with SY, might be useful for further improvement of the selenium intake and status among the Serbian population.

## Figures and Tables

**Table 1:** Sample characteristics by quartiles of plasma selenium.

		Quartile of Plasma Selenium				
	Overall	First	Second	Third	Fourth	P
*n*	142	35	36	36	35	
Age	41.2 ± 8.4	41.0 ± 6.7	39.6 ± 8.6	42.3 ± 8.5	41.8 ± 9.6	0.621
Gender, % male	43.0	31.1	50.0	47.2	42.9	0.411
BMI ^1^, kg/m^2^	26.6 ± 4.0	26.8 ± 4.5	26.0 ± 3.7	26.9 ± 4.1	26.6 ± 3.5	0.916
% of smokers	23.94	25.71	33.33	16.67	20	0.372
% of higher education	59.2	65.7	52.8	58.3	60	0.709
% use of supplements ^2^	23.9	25.7	33.3	16.7	20	0.372
Meat servings/week	4.7 ± 2.1	4.0 ± 2.1 a	4.2 ± 1.9 a,b	4.9 ± 2.0 b	6.0 ± 2.0 c	0.000
Eggs servings/week	1.0 ± 1.1	0.8 ± 0.9	0.9 ± 0.9	1.1 ± 1.1	1.3 ± 1.4	0.357
Milk servings/week	5.2 ± 2.8	5.5 ± 3.2	5.2 ± 2.7	4.7 ± 2.4	5.4 ± 3.1	0.713
Fish servings/week	1.1 ± 1.2	1.2 ± 1.1	1.3 ± 1.4	1.2 ± 1.1	1.3 ± 1.4	0.282
Plasma selenium µg/L	84.3 ± 15.9	67.5 ± 3.15 a	76.9 ± 2.8 b	86.5 ± 3.2 c	107.0 ± 10.0 d	0.000

± Mean and standard deviation, ^1^ Body Mass Index, ^2^ use of vitamin and mineral supplements, values within a row with different letters differ significantly.

**Table 2:** Frequency distribution of food intake in study population and correlation of food intake with plasma selenium.

	Frequency Distribution of Intake (Times/Week)					Correlation	
	<1	1–2	3–4	5–6	≥7	r_s_^2^	p
Meat	0.7% ^1^	8.4%	38.15	38.8%	14.0%	0.369	0.000
Eggs	37.3%	47.8%	14.1%	0.7%	-	0.149	0.122
Milk and dairy	2.8%	12.3%	23.2%	37.3%	23.1%	0.009	0.919
Fish	36.6%	37.3%	14.1%	1.4%	-	0.101	0.149
Meat + eggs	0.7%	8.0%	28.8%	26.1%	37.3%	0.392	0.000
Meat + eggs + fish	0.7%	1.4%	16.2%	26.1%	55.6%	0.437	0.000

^1^ % of study population, ^2^ Spearman’s rank correlation coefficient.

**Table 3:** Comparison of Serbian selenium intake by animal foodstuffs in 1991 and 2013.

	In 1991 [9]		In 2013 [7]	
Foodstuff	Se Concentration	µg Se/Day/Person	Se Concentration	µg Se/Day/Person
Milk	12.2 µg/L	5.0 ^1^	12.1 µg/L	5.9 ^1^
Pork	85.6 µg/kg	4.1	112.7 µg/kg	9.4
Beef	92.0 µg/kg	2.5	95.7 µg/kg	1.7
Poultry	122.4 µg/kg	3.9	121.7 µg/kg	8.0
Eggs	138.0 µg/kg	3.0	186.1 µg/kg	6.7

^1^ calculated as milk and dairy.
